# A single center pilot study: assessing resident needs and faculty perceptions to improve training in rheumatology

**DOI:** 10.1186/s12909-023-04336-8

**Published:** 2023-05-19

**Authors:** Lauren He, Didem Saygin, David Leverenz, Laarni Quimson, Shannon K. Martin, Kichul Ko

**Affiliations:** 1 Department of Medicine, University of Chicago Medical Center, Chicago, IL USA; 2 University of Chicago Section of Rheumatology, Chicago, IL USA; 3 Department of Medicine, Division of Rheumatology and Immunology, Duke University School of Medicine, Durham, NC USA; 4 Univeristy of Chicago Pritzker School of Medicine, Chicago, IL USA

**Keywords:** Medical education, General rheumatology, Survey

## Abstract

**Background:**

Internal medicine (IM) residents lack confidence in rheumatology. Due to the wide variety of topics in rheumatology, identifying the most important subjects to learn during training is vital to create future interventions to increase confidence and knowledge. The preferred teaching modality for both attendings/fellows and residents is not known.

**Methods:**

An electronic survey was distributed to all IM residents, rheumatology fellows, and rheumatology faculty at the University of Chicago during the 2020–2021 academic year. Residents reported self-confidence levels on 10 rheumatology topics, while rheumatology attendings/fellows were asked to rank these from most to least important to learn during IM residency. All groups were asked preferred teaching modality.

**Results:**

Median confidence level [interquartile range] among residents for caring for patients with rheumatological conditions was 6 [3.6–7.5] for inpatient and 5 [3.7–6.5] for outpatient settings (10 being very confident). Attendings and fellows identified the most important topics to learn during the rheumatology rotation as ordering and interpreting autoimmune serologies and musculoskeletal exam. Both attendings/fellows and residents preferred bedside teaching in the inpatient setting and case-based learning in the outpatient setting.

**Conclusions:**

While some disease-specific topics such as autoimmune serologies were identified as important rheumatology topics for IM residents to learn, more practical topics like musculoskeletal exam skills were also deemed important. This highlights the need for comprehensive interventions that focus on more than standardized exam topics alone to improve rheumatology confidence in IM residents. There are different preferences of teaching styles in various clinical settings.

**Supplementary Information:**

The online version contains supplementary material available at 10.1186/s12909-023-04336-8.

## Background

The goal of the rheumatology rotation during Internal Medicine (IM) residency is to develop competency in work-up and treatment of common rheumatologic conditions. Previous studies have suggested that primary care physicians do not feel comfortable diagnosing and managing rheumatologic diseases, with only one third of providers reporting being very confident in co-managing patients with rheumatoid arthritis (RA) despite the majority of providers treating these patients [1]. Internal Medicine (IM) residents report lower self-confidence in rheumatology skills compared to other subspecialties, suggesting the target timing for intervention to increase providers' confidence levels in rheumatology should be during general residency training [2]. Rheumatology is not part of the core inpatient training based on ACGME requirements, therefore the amount of time each resident spends training in rheumatology varies but may be less than other subspecialties. The current study was conducted at the University of Chicago, which does not have a minimal requirement for rheumatology experience during residency. While two-week consult blocks and subspecialty clinics (approximately 8 h every 6 weeks) are available, not all residents complete these. Given less exposure during training, assessment of confidence and competency of trainees in rheumatology becomes important for future internists who will care for patients with these diseases.

There have been previous needs assessments within IM training conducted at other institutions. Leverenz et al. found that resident confidence is low amongst resident learners across most disease categories in rheumatology, yet perceived proficiency by rheumatology educators is typically even lower [3]. Kroop et al. found that IM residents at a single US academic center were not confident in the diagnostic and therapeutic skills required to care for patients with rheumatologic needs, however confidence did increase with increasing post-graduate year [4]. Other needs assessments in rheumatology learning typically focused on an educational intervention (lecture) to improve resident knowledge via comparison of a pre- and post-test scores [5]. However, these interventions did not have resident input and therefore may not be well-suited to meet their specific needs. Additionally, there has been no previous assessment studying if confidence differs among different practice settings. While interventions have been developed to improve rheumatology curricula, most have focused on improving teaching skills of fellows instead of other trainees [6, 7].

As generational preferences in learning style evolve, more emphasis has been placed on tailoring medical education to fit needs of the learner and optimize teaching in an increasingly complex environment [8, 9]. “Learner style” has become an important term in identifying the preferred approach to acquiring new knowledge. While there is a lack of evidence to suggest “traditional” learner styles (visual, auditory, kinesthetic) must match teaching style to achieve improved outcomes, evidence exists showing the importance of ascertaining preferred setting for both learner and teacher within medical education [10]. For example, use of patient simulation as a teaching tool was born from identifying learners' preference for safe, hands-on clinical experience [11]. Learner’s preferences thus have the ability to shift traditional pedagogy in medical education.

The current study seeks to uncover resident learning needs within the field of rheumatology, as well as attending perception of needs, in both inpatient and outpatient settings. We also identify preferred learning platforms for future use. Our objectives were 1) identify the most important topics to learn in the inpatient and outpatient rheumatology rotations based opinions of rheumatology attendings/fellows, 2) assess confidence level of residents in caring for patient with rheumatic conditions in different practice settings, and 3) identify the preferred teaching modalities by rheumatology attendings/fellows and residents in the inpatient and outpatient settings.

## Method

A survey was distributed to all current IM (including IM-pediatrics) residents at the University of Chicago and rheumatology attendings and fellows in the Section of Rheumatology via email. Study data were collected using Research Electronic Data Capture (REDCap) electronic data capture tools hosted at the University of Chicago [12, 13]. REDCap is a secure, web-based software platform designed to support data capture for research studies. Inclusion criteria included 1) currently practicing as a resident, faculty, or fellow at the University of Chicago, and 2) actively providing patient care more than 10% of the time. Exclusion criteria included 1) currently not practicing in the roles mentioned above, or 2) no longer providing active patient care.

Separate attending/fellow and resident surveys were created (Supplemental materials). No validated instruments were available that met our research objectives, therefore new surveys were created and used in this study. Surveys were pilot tested with a small number of participants and adjustments were made based on feedback to improve clarity and accuracy of each survey. Rheumatology attendings/fellows were asked to rank 10 rheumatology topics from most to least important to learn during IM residency. Six topics were based on the American Board of Internal Medicine (ABIM) Certification Exam, and included the following: crystalline arthropathies, rheumatoid arthritis, systemic lupus erythematosus (SLE), spondyloarthropathies, vasculitis, and other ANA-associated diseases (i.e. Sjogren’s, scleroderma) [14]. Appropriate ordering and interpreting of autoimmune serologies, comprehensive musculoskeletal exam, localized joint syndromes, and joint injections were added based on attending and fellow opinion.

The resident survey assessed confidence in caring for patients with rheumatologic conditions in inpatient and outpatient settings on a 10-point Likert scale. Residents were asked their level of confidence (0 = not at all confident, 10 = very confident,) for each of the 10 rheumatology topics described above. Both attendings/fellows and residents were asked preference of teaching modality in the inpatient (bedside teaching, formal lecture, question-based review, providing resources for independent learning) and outpatient setting (case-based learning during clinic, case-based learning after clinic, formal lecture, providing resources for independent learning).

Statistical analysis was completed using IBM SPSS [15]. Descriptive statistics were calculated including average, standard deviation, and interquartile range. Statistical significance was determined using the independent samples t-test for comparison between means. Pearson’s correlation coefficient with t-distribution of the test statistic to determine significance (t = r√(1 − r2)/(N − 2)) was used for correlation between confidence and other parameters. The University of Chicago Institutional Review Board deemed this study exempt due to its status as a survey based educational study; the study has therefore been performed in accordance with the ethical standards in the 1964 Declaration of Helsinki and its later amendments.

## Results

Out of 13 rheumatology attendings/fellows, 11 (84%) completed the survey, including 7 attendings and 4 fellows. Out of 124 residents, 39 (31.5%) completed the survey (Table 1). This included 10 postgraduate year (PGY)-1 residents, 13 PGY-2, 11 PGY-3, 3 PGY-4 and 2 PGY-5. Among respondents, 13 (37%) had completed a rheumatology rotation in medical school and 23 (66%) in residency. Four residents (11%) were interested in rheumatology fellowship. 30 (77%) of residents indicated they were either moderately interested or very interested in learning more about rheumatology.

**Table 1:** Demographics

Total number of resident participants	39	
Gender	Male	21
	Female	18
Training Year	PGY-1	10
	PGY-2	13
	PGY-3	11
	PGY-4	3
	PGY-5	2
Participated in a rheumatology rotation during medical school	24	
Participated in a rheumatology rotation during residency	25	
Average number of weeks of rheumatology service completed	3.2	

**Fig. 1:**
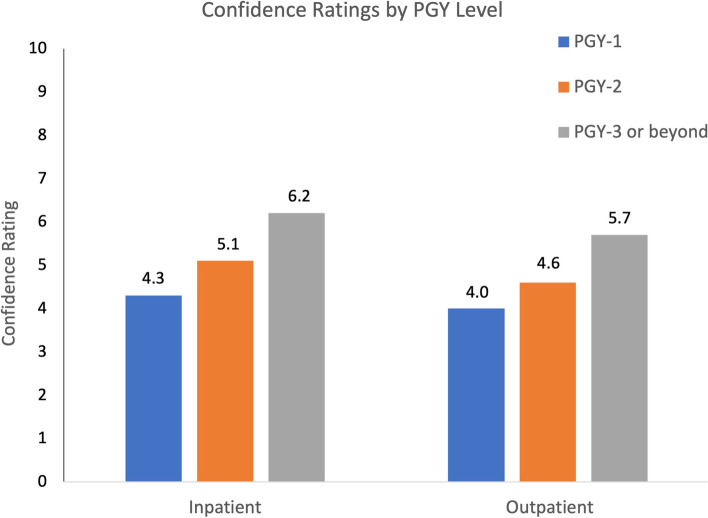
Confidence Ratings by PGY Level. Confidence in managing a patient with rheumatic disease increased with increase training (0 = not at all confident, 10 = very confident). This trend was observed in both inpatient and outpatient settings

Median confidence level [interquartile range] among residents for caring for patients with rheumatologic conditions was 6 [3.6–7.5] in inpatient and 5 [3.7–6.5] in outpatient settings. Confidence levels trended higher with increasing PGY level, but this was not statistically significant (Fig. 1).

**Fig. 2:**
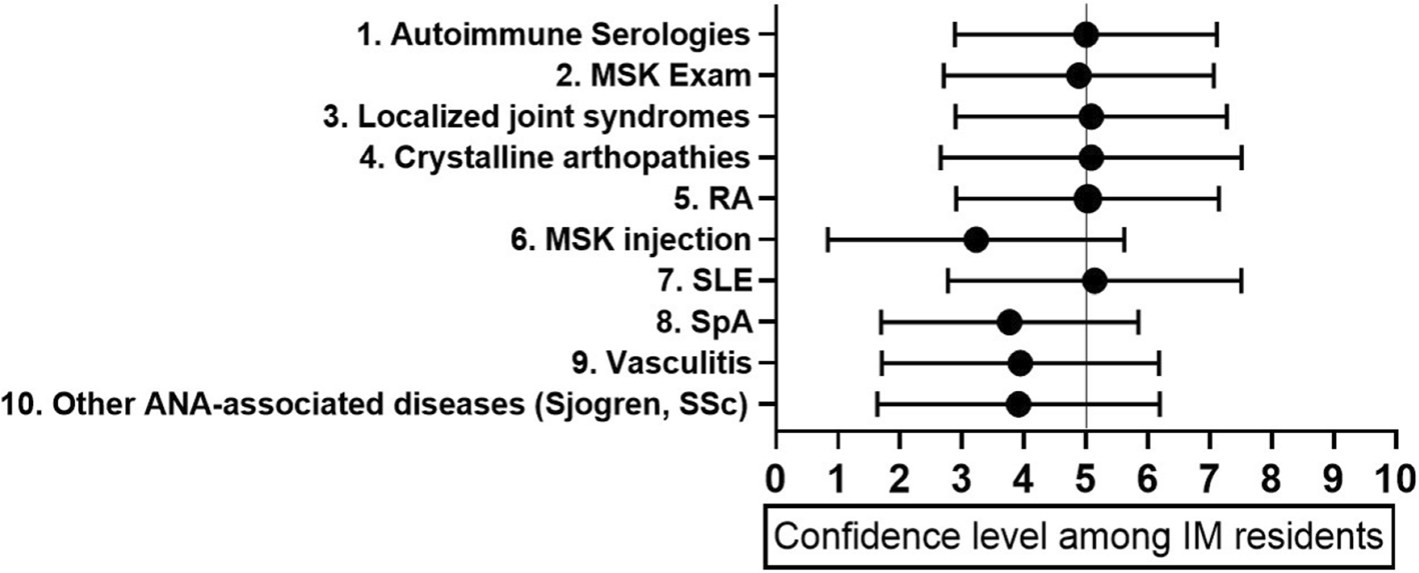
Rank of rheumatology topics and resident confidence levels. Topics listed from most important (1) to least important (10) for IM residents to learn. Resident self-reported confidence level with IQR for each topic is included

**Fig. 3:**
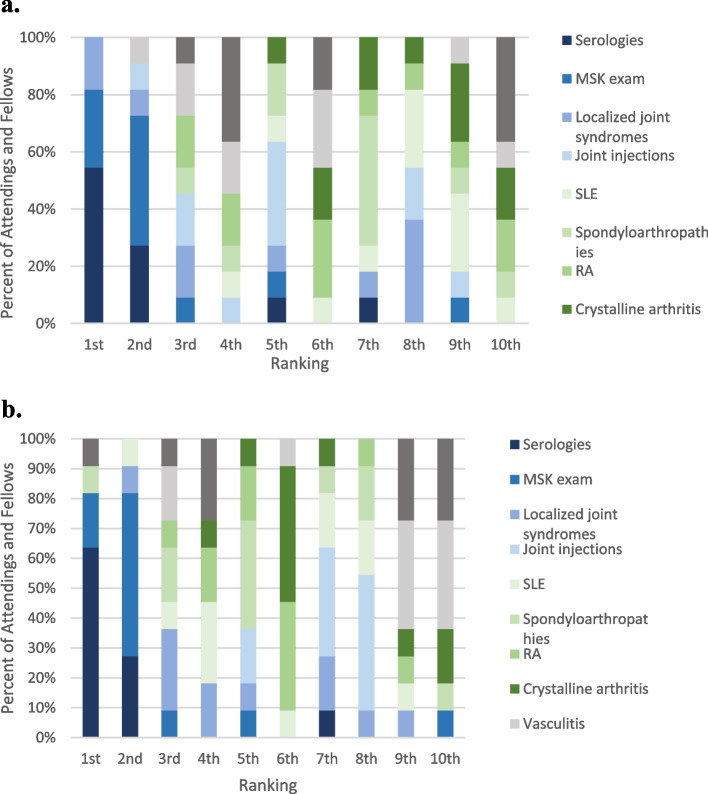
**a, b** Inpatient Ranking Importance of Topics. Knowing when to order serologies and MSK exam were ranked as the most important topics for residents to learn in both the inpatient and outpatient settings by attendings and fellows. **b** In both the inpatient and outpatient settings, attendings/fellows and residents were in agreement with most preferred teaching modality (Fig. 4)

Residents who participated in a rheumatology rotation in medical school were more confident in caring for a patient with rheumatic disease in outpatient clinic than those who did not (5.8 ± 1.8, 4.4 ± 1.9, *p* = 0.031). When compared to residents who had no rheumatology experience in residency, residents who participated in a rheumatology rotation during residency showed no difference in confidence in caring for rheumatology patients on an inpatient (5.6 ± 2.2, 4.9 ± 2.4) or outpatient (5.3 ± 1.9, 4.3 ± 2.1) service. Total number weeks spent on rheumatology services was positively correlated with resident confidence in outpatient (*r* = 0.41, *p* = 0.04) but not with inpatient management (*r* = 0.28, *p* = 0.17). Topics with the least reported confidence included joint injections, spondylarthritis, and other ANA-associated disorders. These topics were ranked 6^th^, 8^th^, and 10^th^ most important to learn during residency by fellows and attendings. Attendings and fellows identified the most important topics to learn during the rheumatology rotation as appropriate ordering and interpreting of autoimmune serologies, followed by musculoskeletal exam and crystalline arthritis (Fig. 2). This was consistent between both inpatient and outpatient settings (Fig. 3a and b).

**Fig. 4:**
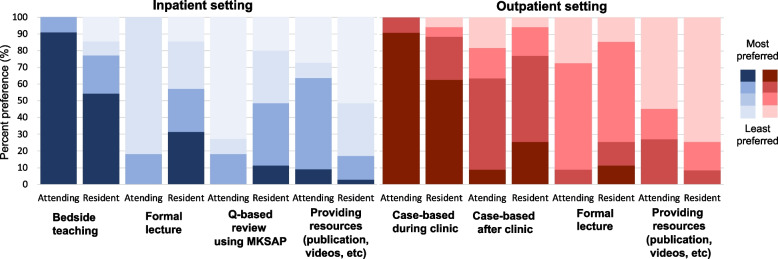
Preferred teaching modality by attendings and residents. Bedside teaching was the preferred modality in the inpatient setting (blue), and case-based learning during clinic was preferred in the outpatient setting (red)

**Fig. 5:**
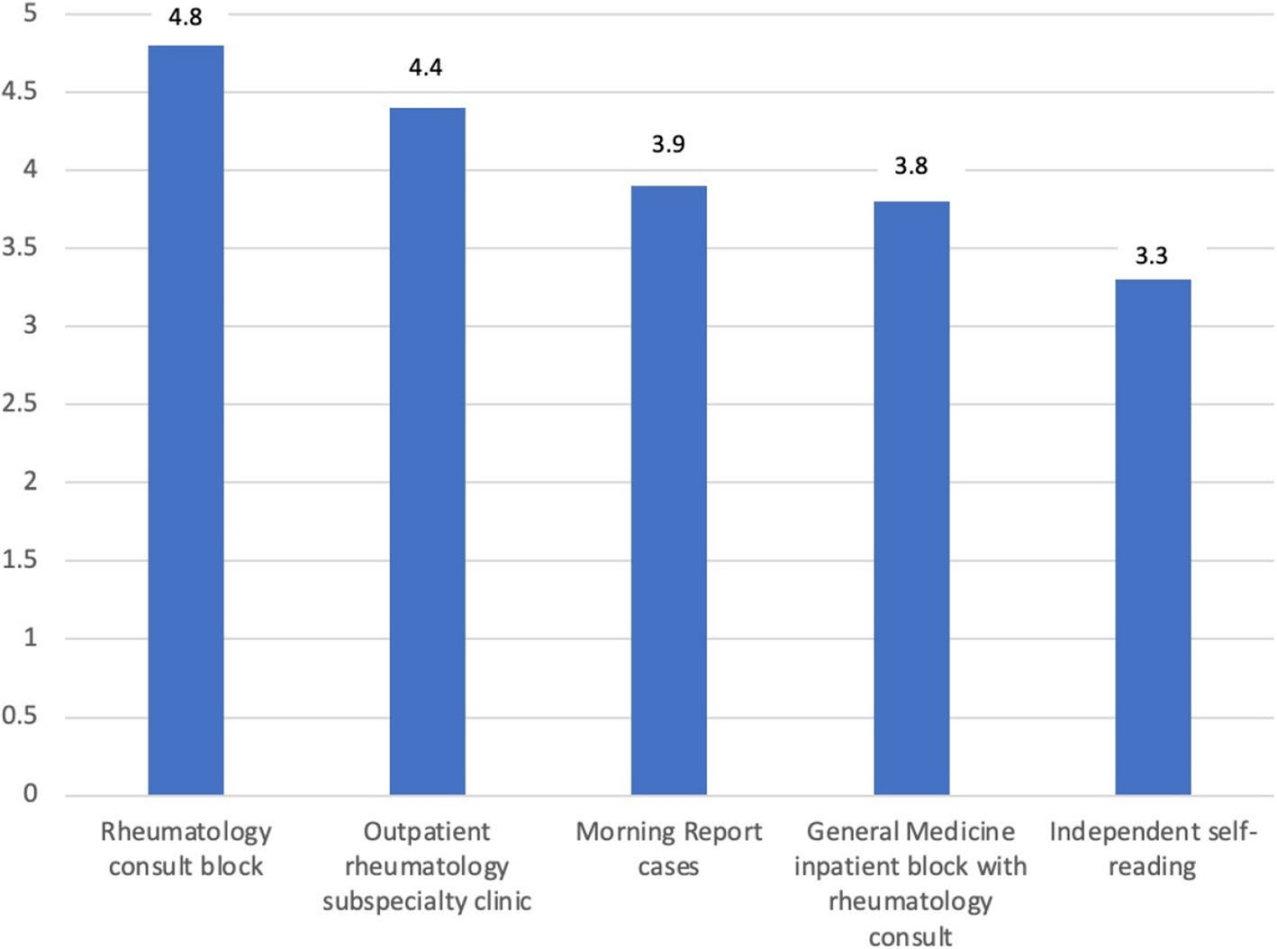
Contribution to resident rheumatology knowledge. Rheumatology consult block was identified as the most helpful from a resident’s perspective to learn rheumatology (5 = extremely helpful), followed by outpatient rheumatology subspecialty clinic

The majority of attendings/fellows and residents preferred bedside teaching in the inpatient setting and case-based learning in the outpatient setting. Formal lectures were more preferred by residents than by attendings/fellows. In contrast, providing resources for independent learning was more preferred by attendings/fellows than residents. In the inpatient setting in particular, providing resources was the 2^nd^ most preferred teaching modality for the majority of attendings/fellows but was least preferred by the majority of residents. Overall, participating as part of the inpatient rheumatology consult team was deemed most helpful by residents, followed by outpatient rheumatology subspecialty clinic (Fig. 5).

## Discussion

Our study showed Internal Medicine residents lack confidence in most core topics of rheumatology, despite many residents having an interest in learning more about the subspecialty. Lowest confidence was reported in MSK injection, spondyloarthropathies, and ANA-associated diseases, all of which were ranked 6^th^ or lower in importance based on attending/fellow opinion. Appropriate ordering and interpretation of autoimmune serologies and comprehensive musculoskeletal exam were deemed the most important topics for residents to be proficient in by fellows and attendings, which was consistent across inpatient and outpatient settings. Regarding learning and teaching preferences, both residents and attendings preferred case-based learning over formal lecture or independent learning in the outpatient setting. In the inpatient setting, bedside teaching was preferred by both residents and attendings, however residents preferred formal lecture next while attendings preferred independent learning.

Residents’ lack of confidence in rheumatology is consistent with previous studies [2–4]. Katz & Oswald (2011) distributed a nation-wide survey to IM residents in Canada and found that residents had significantly lower confidence levels in rheumatology compared to gastroenterology, cardiology, and pulmonology [2]. Importantly, self-confidence in rheumatology improved with increased dedicated teaching rather than years of experience. Our study showed a trend of increasing confidence with increasing PGY level, yet this was not statistically significant. However, residents who had more weeks of experience in rheumatology rotations did have higher confidence in caring for rheumatology patients in the outpatient setting. This interestingly was not the case for confidence with inpatient management, supporting the idea that more experience alone is not sufficient to increase learner confidence. Identifying the teaching modality most preferred by leaners and instructors becomes the next step needed to determine future curricular change to improve resident confidence.

Our study suggests that formal rheumatology teaching should be done via case-based learning in the outpatient setting and bedside teaching in the inpatient setting, as preferred by both learners and teachers. Previous studies have assessed predominant learning styles in residency, however few have looked at preferred learning settings [16, 17]. Gonzalo et al. assessed the learning preferences of IM residents and students and found that learners did not prefer bedside teaching, however the majority did believe that bedside rounds were important for learning core clinical skills [18]. This contrasts with Canton et al. who assessed resident’s most valuable learning experiences, and one of the five most commonly identified themes was bedside learning [19]. Our results show a preference towards bedside teaching in the inpatient setting, which is consistent with both learner and patient preferences at other institutions [20]. Given case based learning was most preferred in the outpatient setting, there is a clear trend towards patient-specific and experience based learning over traditional didactics. This is further supported by residents identifying rheumatology consults rotation, clinic, and morning report cases as most helpful in increasing knowledge. These findings suggest that emphasizing experiential learning and case based didactics may yield the largest benefit in confidence in the care for patient with rheumatic disease.

The impact of increasing confidence in rheumatology training on objective measures such as Board Examinations or In-Training Exam (ITE) scores has yet to be established [4]. Our study identified more practical skills as important for learners, suggesting that emphasis should not be placed on examination scores alone to assess competency. Professional examinations developed by the National Board of Medical Examiners are reflective of current expert opinion in each field but may not encompass all topics that practicing physicians find most important. In fact, our assessment revealed that being able to perform a comprehensive musculoskeletal exam is one of the most important topics for an IM resident to learn. This is not an explicit topic tested on ITE or Board examinations, which highlights that resident curricula designed with core testing topics only may be missing additional clinically important topics.

Strengths of this study include multiple levels of learners and teachers involved (resident, fellow, and attending), as previous needs assessments have not captured learner’s preferences. Comparing resident confidence levels in each category to attending rankings of importance has not previously been assessed, and it is helpful to clarify that topics with lowest confidence were identified as being of lower importance by attendings/fellows. Identifying differences in learning and teaching preferences in both inpatient and outpatient settings has also not previously been established. Limitations of this study include the single-center approach with a small number of participants and a low trainee response rate. The confidence scale was also not assessed in other subspecialty rotations, making it difficult to have a benchmark of where the appropriate level at different times in training should be. Lack of a validated survey assessment also may limit the generalizability of the results. While many trainees indicated they are interested in learning more about rheumatology (which may elicit bias), it is notable that only 1–2 residents each year enter this field from this institution.

## Conclusion

To our knowledge, this is the first study to provide insight into the gaps of rheumatology learning identified by residents and their preferred learning methods to fill these gaps. Internal medicine residents lack confidence across topics in rheumatology, however many have a desire to learn more about the field. Rheumatology attendings and fellows identified the most important topics for residents to learn during training as ordering and interpreting autoimmune serologies and the comprehensive musculoskeletal exam, highlighting the importance of teaching more practical skills which are not fully assessed by standardized examinations. The most desired modalities for teaching these topics are case-based in the outpatient setting and bedside teaching in the inpatient setting. Given this was a single-center pilot study, expanding this survey to other institutions would improve the accuracy and generalizability of results. This survey also allows flexibility in tailoring teaching modalities at different institutions if preferences are found to differ by location. Information gathered using this framework can be used to determine trainee confidence in rheumatology (or any subspecialty) and highlight opportunities to improve existing curricula. These results may help guide more effective inpatient and outpatient teaching strategies in rheumatology, as informed – for the first time – by learner and teacher preferences.

## Supplementary Information

**Additional file 1.**

## Data Availability

The datasets used and analyzed during the current study are available from the corresponding author on reasonable request.
