# Supplementary material for: A single center pilot study: assessing resident needs and faculty perceptions to improve training in rheumatology
Source: BMC Med Educ. 2023 May 19;23:351. doi: 10.1186/s12909-023-04336-8 (PMC10199567; doi:10.1186/s12909-023-04336-8)
Supplement: Supplementary file 1 — Additional file 1. [file 12909_2023_4336_MOESM1_ESM.docx]

A single center pilot study: assessing resident needs and faculty perceptions to improve training in rheumatology

Supplementary Material

Rheumatology Attending/fellow Survey

*Participation in this survey is voluntary and for research use only. The Institutional Review Board of the University of Chicago has reviewed the IRB protocol and deemed it exempt from Human Subjects Review. By continuing to the survey, you are consenting to the use of your answers for research purposes and for future curriculum development.*

1. Which of the following best describes your position?
   1. Rheumatology fellow
   2. Rheumatology attending
2. Which of the following do you identify with?
   1. Female
   2. Male
   3. Non-binary
   4. Prefer not to disclose
   5. Prefer to self-describe (….)
3. If option for attending was chosen in question #1: how many years have you been an attending physician?
   1. 1-5 years
   2. 6-10 years
   3. 11-15 years
   4. 16-20 years
   5. Over 20 years
4. The goal of the inpatient rheumatology rotation during internal medicine residency is to develop competency in recognizing and initiation of basic work-up and treatment of common rheumatological conditions.

Which of the following rheumatology topics do you think is *most important for internal medicine residents* to learn ***during their rheumatology inpatient rotation***? Please rank your choices from 1 (most important) to 10 (least important).

1. Knowing when to order autoimmune serologies and how to interpret the results
2. Musculoskeletal exam
3. Localized joint syndromes
4. Systemic lupus erythematosus
5. Musculoskeletal injections
6. Spondyloarthropathies
7. Rheumatoid arthritis
8. Crystalline arthritis
9. Vasculitis
10. Other ANA-associated diseases (Sjogren’s Syndrome, Mixed Connective Tissue Disease, Systemic Sclerosis)
11. The goal of the outpatient rheumatology rotation during internal medicine residency is to develop competency in recognizing and initiation of basic work-up and treatment of common rheumatological conditions.

Which of the following rheumatology topics do you think is *most important for internal medicine residents* to learn ***during their rheumatology outpatient rotation?*** Please rank your choices from 1 (most important) to 10 (least important).

1. Knowing when to order autoimmune serologies and how to interpret the results
2. Musculoskeletal exam
3. Localized joint syndromes
4. Systemic lupus erythematosus
5. Musculoskeletal injections
6. Spondyloarthropathies
7. Rheumatoid arthritis
8. Crystalline arthritis
9. Vasculitis
10. Other ANA-associated diseases (Sjogren’s Syndrome, Mixed Connective Tissue Disease, Systemic Sclerosis)
11. What additional topics do you think internal medicine residents should learn during training (other than the topics listed in the previous questions)?
12. What type of learning platform do you prefer to use while teaching rheumatology topics to residents ***in the outpatient setting?*** Please rank your choices from 1 (most preferred) to 4 (least preferred).
    1. Case-based teaching while a resident is staffing with you
    2. Discussing cases seen in clinic after the clinic is completed
    3. Formal didactic lecture outside of clinic
    4. Directing trainees to relevant resources for self-learning (papers, review articles, videos, etc.)
13. What type of learning platform do you prefer to use while teaching rheumatology topics to residents ***in the inpatient setting*?** Please rank your choices from 1 (most preferred) to 4 (least preferred).
    1. Bedside teaching
    2. Formal lecture
    3. Question-based review using MKSAP
    4. Directing trainees to relevant resources for self-learning (papers, review articles, videos, etc.)
14. For positive ANA referrals in the outpatient setting, how often do you feel your patients are informed by their PCP about the non-specificity of the test?
    1. Always (close to 100% of the time)
    2. Most of the time (>75% of the time)
    3. More than half of the time (50-75%)
    4. Less than half of the time (25-50%)
    5. Rarely (<25%)
    6. Never

Resident Survey

*Consent statement: Participation in this survey is voluntary and for research use only. The Institutional Review Board of the University of Chicago has reviewed the IRB protocol and deemed it exempt from Human Subjects Review. By continuing to the survey, you are consenting to the use of your answers for research purposes and for future curriculum development.*

Demographics

- - - 1. What is your current PGY year?

PGY-1

PGY-2

PGY-3

PGY-4

PGY-5 or beyond

1. Which of the following do you identify with?
   1. Female
   2. Male
   3. Non-binary
   4. Prefer not to disclose
   5. Prefer to self-describe (….)
2. Which of the following fields are you planning on pursuing?
   1. Allergy/Immunology
   2. Cardiology
   3. Endocrinology
   4. Gastroenterology
   5. General Medicine, Hospitalist, or Primary Care
   6. Geriatrics
   7. Hematology/Oncology
   8. Infectious Diseases
   9. Nephrology
   10. Palliative Care
   11. Pulmonology/Critical Care
   12. Rheumatology
   13. Sleep medicine
   14. Sports medicine
   15. Not sure
3. Did you participate in a rheumatology rotation in medical school?
   1. Yes: outpatient clinic
   2. Yes: inpatient consults
   3. Yes: both outpatient clinic and inpatient consults
   4. No
4. Did you participate in a rheumatology rotation so far during your residency?
   1. Yes: outpatient
   2. Yes: inpatient consults
   3. Yes: both outpatient and inpatient consults
   4. No
5. If yes; how many weeks of inpatient consult or outpatient rotation in rheumatology have you had so far in total? Free text answer (---)
6. What is your level of interest in learning about topics related to rheumatology?
7. Not at all interested
8. Moderately disinterested
9. Nether disinterested or interested
10. Moderately interested
11. Very interested

Confidence Assessment

1. The goal of the inpatient rheumatology rotation during internal medicine residency is to develop competency in recognizing and initiation of basic work-up and treatment of common rheumatological conditions.

How confident do you feel caring for a patient with an active rheumatologic diagnosis (related to their reason for admission) ***on the general medicine service?***

| 1 | 2 | 3 | 4 | 5 | 6 | 7 | 8 | 9 | 10 |
| --- | --- | --- | --- | --- | --- | --- | --- | --- | --- |
| **Not at all confident**  I completely rely on the consulting rheumatologist to guide initial diagnostic workup and treatment for patients with rheumatologic disease *in inpatient practice*. |  |  |  |  |  |  |  |  | **Very confident**  I can independently recognize and initiate basic workup and treatment for patients with rheumatologic disease *in inpatient practice*. |

1. The goal of the outpatient rheumatology rotation during internal medicine residency is to develop competency in recognizing and initiation of basic work-up and treatment of common rheumatological conditions.

How confident do you feel caring for a patient with a rheumatologic diagnosis ***in primary care clinic*?**

| 1 | 2 | 3 | 4 | 5 | 6 | 7 | 8 | 9 | 10 |
| --- | --- | --- | --- | --- | --- | --- | --- | --- | --- |
| **Not at all confident**  I am not confident that I could recognize a rheumatologic condition or initiate basic workup and treatment for patients with rheumatological disease *in outpatient practice*. |  |  |  |  |  |  |  |  | **Very confident**  I can independently recognize and initiate basic workup and treatment for patients with rheumatologic disease *in outpatient practice.* |

1. How much did each of the following contributed to your knowledge in rheumatology? Please choose from 1 (not at all helpful) to 5 (extremely helpful). Please select “not applicable” if you did not complete or participate in an option.
2. Rheumatology consult block
3. General Medicine Inpatient block with rheumatology consult for your patients
4. Outpatient rheumatology subspecialty clinic
5. Morning report
6. Independent self-reading
7. ***How confident*** do you feel in regards to clinical knowledge about each of the following topics? (0=not at all confident, 10=very confident)
   1. Knowing when to order autoimmune serologies and how to interpret the results
   2. Musculoskeletal exam
   3. Localized joint syndromes
   4. Systemic lupus erythematosus
   5. Musculoskeletal injections
   6. Spondyloarthropathies
   7. Rheumatoid arthritis
   8. Crystalline arthritis
   9. Vasculitis
   10. Other ANA-associated diseases (Sjogren’s Syndrome, Mixed Connective Tissue Disease, Systemic Sclerosis)
8. What type of learning platform would you prefer to learn more about rheumatologic topics in the ***inpatient setting*?** Please rank from 1 (most preferred) to 4 (least preferred).
   1. Bedside teaching
   2. Formal lecture
   3. Question-based review using MKSAP
   4. Being given relevant resources from attendings and fellows for self-learning (papers, review articles, videos, etc.)
9. What type of learning platform would you prefer to learn more about rheumatologic topics in the ***outpatient setting***? Please rank from 1 (most preferred) to 4 (least preferred).
   1. Case-based learning while staffing a patient with an attending
   2. Discussing cases seen in clinic after the clinic is completed
   3. Formal didactic lectures outside of clinic
   4. Being directed to relevant resources by attending for self-learning (papers, review articles, videos, etc.)
10. When you order an anti-nuclear antibody (ANA) in the primary care setting, how often do you counsel your patients on the non-specificity of the test?
    1. Always (close to 100% of the time)
    2. Most of the time (>75% of the time)
    3. More than half of the time (50-75%)
    4. Less than half of the time (25-50%)
    5. Rarely (<25%)
    6. Never
    7. Unaware of the non-specificity
